# A new eye lens structure associated with capsule/basement membrane growth.

**DOI:** 10.17912/micropub.biology.000828

**Published:** 2024-07-29

**Authors:** Wouterus TM Gruijters

**Affiliations:** 1 Holy Cross Seminary, Auckland, New Zealand

## Abstract

Eye lens capsules contained a previously overlooked structure possibly derived from the Zonule of Zinn or lens epithelium. Sheep lens capsule inner surface revealed periodic stripes spaced at about 8µm over an area of more than 200µm long and wide. Ultra thin sections of a similar area revealed the periodic insertion of cell feet into the lens capsule with numerous vesicles. Cryosections of entire mouse eyes confirmed a similar looking structure. The structure appears in an area of basement membrane growth. A stylized foot-feet model is offered to help visualize the structure in 3D.

**Figure 1. Images of a previously undescribed structure in the eye lens:**
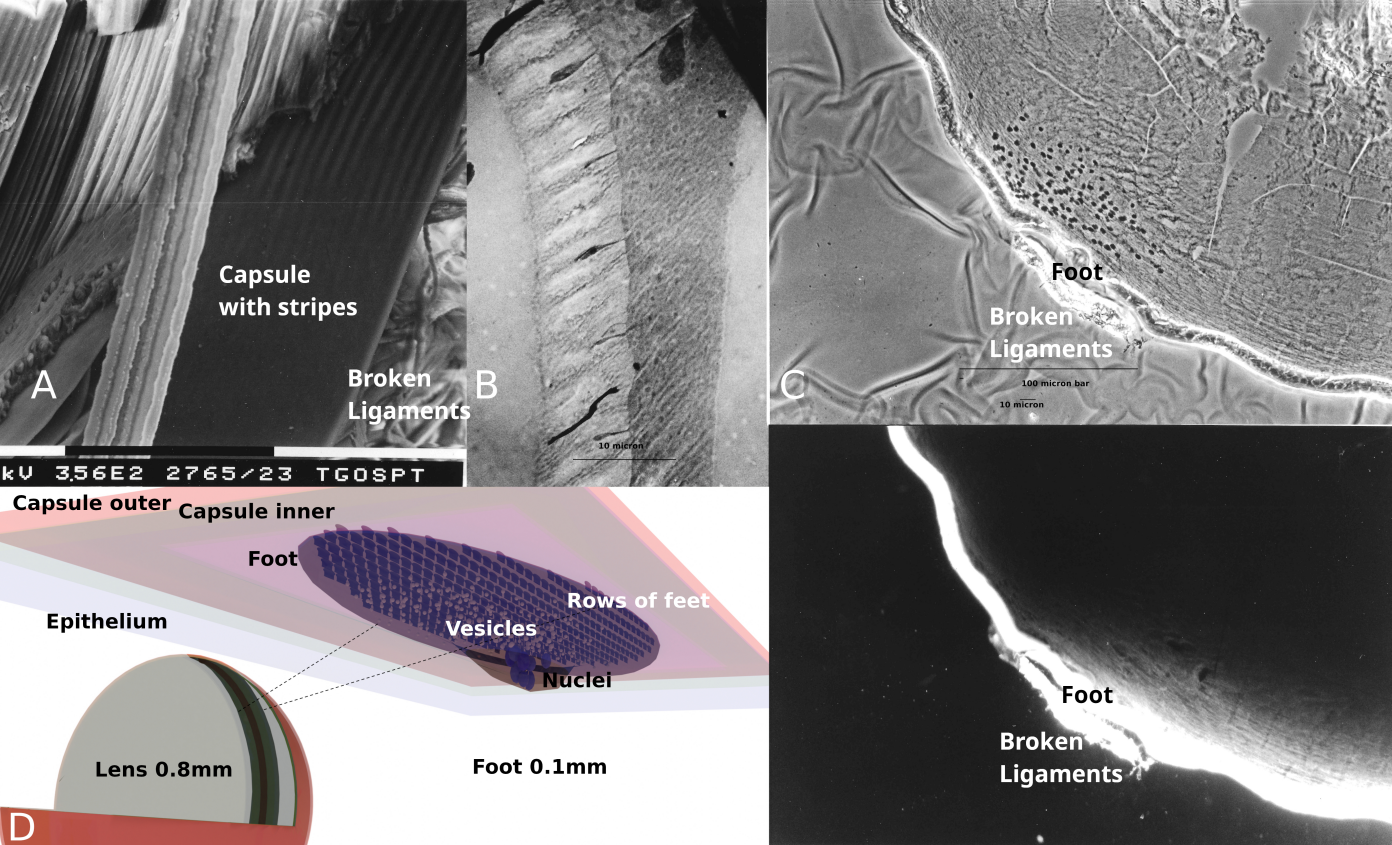
(A) Scanning electron micrograph of a micro-dissected sheep lens from a lamb less than a year old. Cells pull apart cleanly with membranes predominantly left intact. The capsule is very thin here, probably less than 2µm, as shown by the sheared edge being rounded but still appearing thin. The striped pattern near where the ligaments attach to the lens were not seen in large areas of well preserved thicker capsule elsewhere in the lens. Magnification bar 100µm (B) Transmission electron micrograph of lens capsule removed from a mouse, fixed and stained with heavy metals prior to ultra-thin sectioning. Top right is a copper supporting grid bar with adjacent darkly stained elongated cell nuclei. Circular white vesicles surround the nuclei and extend to the left and down into a band of the grayer cytoplasm. Further to the left is a nominally 10µm thick more lightly stained basement membrane with cytoplasmic feet and fibrils extending into and through it. (C) Interference contrast microscopy above and FITC coupled WGA fluorescence microscopy below can show structural features that parallel structures seen with electron microscopy. (D) A 3D highly stylized foot-feet model of basement membrane growth in a nominally 0.8mm diameter spherical 8 week old mouse lens based on Fig. 1C. The cells may form the foot from either the epithelium or the cells migrating along the ligaments towards the lens surface where they move to areas where they are needed. Blue spheres represent nuclei inside a cell. Smaller white spheres represent vesicles. The rows of numerous feet are highlighted by making them darker than the surrounding gray foot.

## Description

Basement membranes have been found to perform a plethora of functions in an array of structures in most tissues, often studied in relation to disease (Jandl et al 2022) . They are defined as highly specialized, thin, acellular extracellular matrices underlying cells that separate them from, as well as connect them to, their associated matrix (Aumailley et al 2005) . Basement membranes vary considerably in function under this definition (Wilson et al 2020) and the eye lens capsule is an extreme example of this variation. While referred to as a basement membrane (Ruben & Yurchenco 1994) , it is often more than 5µm thick, with an area measured in square millimeters (Fisher 1969) , dwarfing adjacent cells. It is required to hold the eye lens in place, and is attached to and suspended by very fine ligaments called the Zonule of Zinn while holding the eye lens in an optically correct configuration at changing focal lengths (Bassnett 2021) . It is the large proportions of this basement membrane that make it easier to dissect and investigate than other basement membranes. A continuous growth model for the cellular portion of lenses is widely accepted in the literature (Liu et al 2020) . In order for the lens capsule to accommodate this continual growth the capsule must also grow but has been difficult to investigate (Shi et al 2013) . Here we interpret micrographs to model a new structure consisting of a cell, sometimes a multi-nuclear physiological syncitia, involving vesicles, fibrils and periodically spaced cytoplasmic feet in the growing region of the capsule. The new structure or parts of it are often seen in appropriately prepared samples, though a good estimate of the number per lens or size variability is not available. It is likely to change over time as the lens gets larger throughout life reducing the surface area to volume ratio. A stylized 3D “foot-feet” model structure of the structure is offered as a starting point for further discussion.

**Interpretation of images**

Screening and comparing hundreds of micrographs from sheep and mouse lens experiments resulted in some that could not be interpreted using only knowledge of known lens structures. Fig. 1A is an example of a lamb eye lens showing a clear pattern of stripes on the surface of the capsule closest to the lens epithelium . Consideration was given to the banding reflecting the organization of epithelial cells, or elongating fiber cells on the other side of the epithelial cells, or cells at the posterior surface of the lens where fiber cells contact the capsule, however no correlation could be found. A continuous pattern of similar stripes could be seen extending around the lens near the equator where the capsule was peeled away. The pattern was superimposed onto the apparent mosaic of the epithelium. The band of stripes is approximately 300µm wide in the sheep lens and would presumably extend for about 3cm around the lens circumference if it were all visible. Similar stripes were not observed anywhere else on the samples. The other notable aspect of this area of the dissected capsule was the cross sectional thickness. Micrographs of capsule edges millimeters away showed clearly defined sheared edges around 10µm thick, whereas the sheared capsule edge in this and adjacent areas was different in appearance. It was typically so thin as to have a rounded or curled rather than sheared edge where it had been dissected making it difficult to assign an accurate thickness. This was partly due to the limitations of tilt in the scanning electron microscope. The correlations between structure and function attributed to this area are the proximity to ligament attachments, possible capsule growth, and alignment of the mosaic of epithelial cells into more regimented rows of fiber cells as they elongate.

Fig. 1B shows an ultra -thin section of a mouse lens capsule pulled off the mouse lens. At the top of the image, next to a black supporting copper mesh, are a few darkly stained nuclei surrounded by smaller white circular vesicles. The nuclei and vesicles are in the darker band of cytoplasm to the center right of the micrograph, while the more lightly stained capsule is to the center left of the micrograph. Nominally the cytoplasm and capsule measures about 10µm thick each in Fig. 1B, however perception of the structure is likely to overestimate the real thickness as the chance of mounting and cutting such a flexible structure perpendicular to the knife cut is remote . Other light micrographs from the mouse eye in this area confirm the actual thickness of the capsule is usually much thinner than 10µm, probably in places less than 3µm, with the resolution of the light microscopy techniques used preventing a more definitive measure.

Also visible in Fig. 1B are club shaped periodic extensions of the cytoplasm into the capsule along with periodic light and dark staining areas of the capsule parallel to these . Such club shaped cell extensions might be classed as pseudopodia. The context, size, highly organized structure and possible functions of the cell extensions in a basement membrane are not characteristic of pseudopodia in the wider literature. There are perhaps greater similarities with the more recently described filopodia, lamipodia, (Arjonen et al 2011, Heckman & Plummer 2013, Jacquemet et al 2015) or perhaps ruffles or spikes (Lynn et al 2020) . However the new structure’s regularity of the pseudopodia over a large area and grouping of cell nuclei, along with the functions they appear to be involved in, is not a very good match for any of these pseudopodia like structures. Here the cytoplasmic extensions will be referred to simply as feet to reflect their even size and periodicity. At higher magnifications the light/dark banding down the capsule in the image can be seen to result from, among other things, higher and lower densities of stained fibrils that may parallel the stripes in Fig. 1A . It is highly likely this banding is the same as that reflected in the scanning electron micrograph images. Still higher magnifications show even finer fibrils appearing within the feet along with an occasional organelle. Also seen at higher magnifications along with the larger vesicles near the nuclei are more numerous smaller vesicle like structures nearer the capsule.

Fig. 1C demonstrates a structure approximately the right size and shape to be the equivalent structures seen in the Fig . 1A&B. It is with rapid fixation and cryoprotection prior to cryosectioning that the structure was seen in newborn mouse lenses. The rapid uptake of WGA into the structure gives some indication of metabolic activity. Unfortunately the resolution of the light microscope images does not allow certainty that this is the same structure seen in electron microscopy. While a probably continuous 100µm wide band of stripes extend around the lens periphery in newborn mice, the structure appears not to be reported by others. One reason for this lack of reporting may be the fragility of the structure which is easily lost during dissection of the eye. Another reason may relate to the structure becoming smaller or less frequent relative to the overall lens as the lens grows. Also related to growth, the surface area to volume ratio decreases the larger the lens gets demanding proportionately less new basement membrane over time.

The discovery of a biological structure that has all the characteristics required for provisioning growth of the lens capsule or aid in fiber organization is represented here in the form of one possible model. It is necessarily provisional in nature. In order to generate the model five major assumptions are made and briefly considered.

**Assumptions**

1) A thin zone of capsule near the anteriorly and posteriorly attached ligaments at the lens equator is the main region for generating new basement membrane and alignment of differentiating epithelial cells into more organized rows of fiber cells. It cannot be discounted that growth of the capsule, particularly thickening, may also occur in other parts of the lens using similar structures without the adjacent suspensory ligaments.

2) Where the cells originate to form the new structure being modeled is unclear. In Fig. 1C the structure appears to form from the aggregation of lens epithelial cells . Migration of cells to the outside of the lens from the inside has not been observed or reported. It is assumed that the structure could also be derived from extra-lenticular cells, perhaps from the ciliary body, something which has been theorized (Remington & Meyer 2007) , perhaps via the suspensory ligaments. It is also possible that cells within the lens also contribute to form the structures on the opposing capsule surface. There has been no literature suggesting the cells cross the capsule though considering the size of the new structure that has been overlooked this cannot be discounted either.

3) Club shaped structures in thin sections reflect a two dimensional profile and are more likely to be elongated feet-like structures although it is unclear how long they are. Scanning electron microscopy of the three dimensional structure gives a good indication of the spacing of the feet. Feet size and shape are derived from thin sections that are essentially two dimensional making the dimensions imprecise.

4) Suspensory ligament attachments to the capsule must also adapt as the lens grows so capsular growth is likely to be associated with the suspensory ligaments at the equator. As actual mechanisms for the process of lens ligament growth during the life of the animal are not yet described in the literature, the assumption is that there must be such a process so this new structure may be involved.

5) While others suggest the equator as a zone of growth (Liu et al 2020) it is unclear whether the new structure is patchy or regular around the lens equator. While light micrographs lack the resolution to give certainty, it is likely to be more patchy in older animals due to the new structure being undescribed so far and only appearing infrequently in micrographs of the area. However, the lack of reports of the new structure could also be due to the fragility of the area during preparation for microscopy. The organized foot print of stripes continuous around the year old sheep lens equator allows the assumption that it may well be continuous.

**The “foot and feet” model of capsular growth**

Fig. 1D shows one possible stylized model of the new structure, perhaps derived from the lens epithelium, in the process of laying down capsule on the lens exterior . Origins of the cell from the ciliary body on the other side of the capsule cannot be discounted. The cells arrive at either the ligament side or the opposing side of the capsule to form a “foot”. In this foot the cells are actively manufacturing basement membrane components on the capsule exterior as reflected by the large number of vesicles and numerous “feet”. Changes in ligament attachments required during equatorial growth would include suspensory ligament material to maintain contact between the lens and zonules. The systematic laying down of new capsular material and fibers into the secreted capsular matrix is facilitated by the periodic presence of smaller feet which the larger foot produces. These feet could aid in the appropriate spacing of structural fibers and precise thickening of the capsule exterior required for it to have the correct elasticity and strength. Precise dimensions and properties of the capsule basement membrane are needed to maintain and systematically change lens shape during the eye focusing. The periodicity of the feet in the structure could also aid in the organization of epithelial cells into rows of fibers. Further from the foot the capsule may continue to grow to its final thickness by provisioning additional basement membrane components from fiber or epithelial cells.

Having a testable model of any new structure is important. Testing in this case will involve others being able to more systematically find, preserve and photograph the structures. That structures exist in the lens with unknown functions is clear from the micrographs presented but further research is needed. Though not referred to in the eye lens literature, the closest looking known structures to the foot-feet structure are probably filopodia. While each single cell extension may bear some resemblance, filipodia lack the regimented size, periodicity, multi-nucleate nature and broad attachment of the overall foot-feet structure. Filopodia may hold clues to the foot-feet roles in the lens and the parallels will be useful. While the structures are correlated with areas of probable basement membrane extension and organization of epithelial cells into rows during differentiation there is no certainty these these are the structure's role. Perhaps foot-feet have an unrelated function such as shifting ligaments, growing or attaching them. Equivalent foot-feet structures may be found in basement membranes elsewhere in the body if other difficult to decipher micrographs are looked at.

## Methods

All micrographs were selected from an archive of photos from from experiments done prior to the year 2000. No additional animals were sacrificed for this paper. For the original published studies (Gruijters 1989, Evans et al 1993, Gruijters 2003) , samples were prepared as soon as possible (usually within minutes) after the death.

**Scanning electron microscopy**

For Fig. 1A freshly sacrificed lambs had the lens removed and placed directly into 2% buffered freshly made formaldehyde for a day or days at most until a firm but not solid texture was reached. Micro-dissection of the lens was with manually sharpened Inox #5 tweezers, followed by additional fixation in 1% osmium tetroxide and 6% glutaraldehyde in a a suitable phosphate buffer. Serial dehydration was in ethanol followed by critical point drying. The dehydrated sample was rotary coated with a carbon platinum mixture by vacuum evaporation. Dissected cells viewed by ultrathin sectioning can me shown to separate cleanly from each other during dissection.

**Transmission electron microscopy**

For Fig. 1B the capsule from a mouse was pulled away from the lens, fixed in 2% glutaraldehyde, serially dehydrated and embedded in resin to allow ultra-thin sectioning. Thin sections were stained with uranyl acetate and lead citrate to enhance contrast with some pooled and precipitated stain being trapped on some parts of the sections. Due to a lack of osmium tetroxide stain the membranes tend to appear light. A good membrane stain would be advisable in future studies.

**Light microscopy**

For Fig 1C an 8 week old mouse eye was extracted and slit to allow penetration of WGA solution within 15 minutes. After 6 minutes in WGA it was then fixed in freshly made 2% PBS formaldehyde for 10 minutes. It was then 11% PBS glycerinated for 2 hours and cryosectioned. Both interference contrast and fluorescent micrographs are of the same area. While glycerin reduces freezing damage in the sample it has the drawbacks of making the lens softer to cut resulting in more distortion and adding to birefringence.

**Modeling and imaging**

3D models were generated using blender for artists https://blenderartists.org/ running under Unbuntu 22.04.2 LTS. Labeling and adjusting contrast on 2D renderings was with ShotWell Viewer. Scanning of micrographs was done with an Officejet Pro 8500 scanner at 600 DPI on default gray level contrast settings. Cropping, sizing, rotating and labeling of micrographs used Libre Office Draw and GIMP with no change in contrast, brightness or any other image editing.
